# Associations between comorbidities and patient-reported outcomes in axial spondyloarthritis: data from a single-center real-world cohort

**DOI:** 10.1007/s10067-025-07452-6

**Published:** 2025-05-07

**Authors:** Grzegorz Biedroń, Mateusz Wilk, Jarosław Nowakowski, Piotr Kuszmiersz, Zofia Guła, Magdalena Strach, Alen Brkic, Glenn Haugeberg, Mariusz Korkosz

**Affiliations:** 1 Department of Rheumatology and Immunology, Jagiellonian University Medical College, 2 Jakubowskiego Street, 31-315 Cracow, Poland; 2 Department of Rheumatology, Clinical Immunology and Internal Medicine, University Hospital in Cracow, Cracow, Poland; 3 Division of Rheumatology, Department of Internal Medicine, Sørlandet Hospital, Kristiansand, Norway; 4 Department of Neuromedicine and Movement Science, Faculty of Medicine and Health Sciences, NTNU, Norwegian University of Science and Technology, Trondheim, Norway

**Keywords:** Axial spondyloarthritis, Comorbidity, Multimorbidity, Patient-reported outcome, Quality of life

## Abstract

**Introduction:**

Comorbidities are frequently present in patients with axial spondyloarthritis (axSpA) and may contribute to poorer health-related outcomes. Patient-reported outcomes (PROs) serve as tools to assess the patient’s perspective on the burden of the disease. The study aimed to evaluating the impact of comorbidities on selected PROs in axSpA.

**Method:**

Adult patients diagnosed with axSpA based on ASAS classification criteria were included in this cross-sectional study. Data collected included comorbidities and PROs [Health Assessment Questionnaire (HAQ), Multi-Dimensional Health Assessment Questionnaire (MDHAQ), 36-Item Short Form Health Survey (SF-36), Bath Ankylosing Spondylitis Disease Activity Index (BASDAI), and Bath Ankylosing Spondylitis Functional Index (BASFI)].

**Results:**

In total, 323 participants were included in the study (44.0% female). Multimorbidity and extended multimorbidity were observed in 63.8% and 38.7% of patients, respectively. Extended multimorbidity was associated with higher HAQ (0.5 [0.1–1.0] vs. 0.3 [0.0–0.8], *p* < 0.01) and MDHAQ scores (0.3 [0.0–0.6] vs. 0.1 [0.0–0.5], *p* = 0.03). The BASDAI score was higher in patients with three or more comorbidities than in patients with no comorbidities (2.8 [1.7–4.3] vs. 1.9 [0.9–3.6], *p* < 0.01). The number of comorbidities was associated with higher scores in the mental health (*β* = 0.20, *p* < 0.01) and vitality (*β* = 0.16, *p* = 0.02) domains of the SF-36 questionnaire.

**Conclusions:**

Multimorbidity was present in almost two-thirds of cases. Extended multimorbidity was associated with poorer physical functioning, HAQ, MDHAQ, and BASDAI scores, whereas multimorbidity was related to better scores in the mental health and vitality domains. The impact of comorbidities on PROs in axSpA should not be overlooked.

**Table:** 

**Key Points** • *Multimorbidity is frequent among axial spondyloarthritis’ patients*. • *Only extended multimorbidity (presence of two or more additional diseases, excluding axial spondyloarthritis) was found to be associated with poorer results of patient-reported outcomes, in particular these regarding physical functioning*. • *Multimorbidity might influence positively some domains of quality of life such as mental health or vitality*. • *The findings included in the study support the belief that in patients with spondyloarthritis comorbidities might impact patient-reported outcomes which are important when assessing disease activity and response to treatment and should not be disregarded*.

**Supplementary Information:**

The online version contains supplementary material available at 10.1007/s10067-025-07452-6.

## Introduction

Spondyloarthritis (SpA) refers to a group of chronic inflammatory diseases of unknown origin with shared pathophysiological and clinical features, primarily affecting the musculoskeletal system (e.g., spine, joints, entheses, and ligaments) [1, 2]. Axial SpA (axSpA) is characterized by predominant involvement of the sacroiliac joints and spine. It encompasses two main classifications: ankylosing spondylitis (AS), which fulfils the radiographic Modified New York criteria [3], and non-radiographic axSpA, which does not show significant X-ray abnormalities but meets the Assessment of Spondyloarthritis International Society (ASAS) criteria [4]. The prevalence of axSpA is estimated to range from 0.3 to 1.4% [5, 6]. Its natural disease progression may lead to significant disability, resulting in a substantial burden for both the affected individual and society [7]. The current standard pharmacological treatment of axSpA mainly includes non-steroidal anti-inflammatory drugs, biologic disease-modifying anti-rheumatic drugs (DMARDs), and targeted synthetic DMARDs [8]. State-of-the-art management requires a treat-to-target strategy, necessitating precise assessment of disease activity [8]. Up-to-date evaluation of patients with axSpA should consider not only objective factors (e.g., number of swollen joints, radiological images, and inflammatory parameters such as C-reactive protein [CRP]) but also patient-reported outcomes (PROs).

PROs are subjective symptoms, emotional states, and other aspects of personal functioning and well-being assessed directly by patients. Different tools for evaluating PROs are commonly used in contemporary rheumatology [9–11]. Several questionnaires have been designed and validated for rheumatic diseases, including SpA. The Health Assessment Questionnaire (HAQ) evaluates a patient’s disability in the following sections: dressing, arising, eating, walking, hygiene, reach, grip, and activities [12]. The Multi-Dimensional Health Assessment Questionnaire (MDHAQ), an extended version of the HAQ, comprises the Multi-Dimensional Health Assessment Questionnaire Functional (MDHAQFn) and the Multi-Dimensional Health Assessment Questionnaire Psychological (MDHAQPs), assessing functional and psychological aspects, respectively [13].

The 36-Item Short Form Health Survey (SF-36) evaluates quality of life across separate domains, including mental health (MH), vitality (VT), bodily pain (BP), general health (GH), social functioning (SF), physical functioning (PF), role limitations due to physical problems (RP), and role limitations due to emotional problems (RE) [14].

The Bath Ankylosing Spondylitis Disease Activity Index (BASDAI) is a tool designed to assess disease activity in AS/axSpA. The index involves self-reported symptoms such as fatigue, stiffness, tenderness, and pain in specific areas of the musculoskeletal system. The score is calculated based on the points assigned to each of six questions using a visual analogue scale (VAS) or numerical rating scale (NRS) [15]. It remains widely used to evaluate treatment efficacy, particularly in support of a treat-to-target strategy. However, the Ankylosing Spondylitis Disease Activity Score (ASDAS), which incorporates laboratory results for inflammatory markers, is preferred for this purpose in accordance with ASAS-EULAR recommendations [8].

Another tool based on patients’ self-assessment, introduced for evaluating outcomes in AS/axSpA, is the Bath Ankylosing Spondylitis Functional Index (BASFI). Its structure is similar to that of the BASDAI, consisting of eight patient-assessed categories using a VAS or NRS. The questions address functionality and the ability to manage everyday life in the context of the disease [16].

Pain catastrophizing is considered an exaggerated perception of pain, combined with rumination and a feeling of helplessness in managing it. It is an important factor influencing patients’ well-being in rheumatic diseases [17].

The introduction of PROs enabled medical professionals to focus more deeply on patients’ perspectives and expectations concerning disease management, which resulted in more effective treatment regimens. However, because these tools are subjective, their interpretation may be less clear in the presence of confounding factors.

Comorbidity, defined as the presence of at least one additional disease with or without a causal link to the primary entity, is common in patients with axSpA. Among the most prevalent comorbidities are hypertension, hypercholesterolemia, osteoporosis, depression, gastrointestinal ulcers, obesity, diabetes mellitus, and chronic pulmonary disease [18–26].

The presence of comorbidities may interfere with the assessment of axSpA activity by influencing patients’ symptoms and signs. Multimorbidity is a term closely related to comorbidity but presents a perspective focused on the patient. It is defined as the presence of two or more chronic diseases in the same individual [27].

The aim of this study is to assess the impact of comorbidities on a wide range of PROs in axSpA.

## Materials and methods

This cross-sectional, observational study was based on real-world data collected in the Department of Rheumatology and Immunology, Jagiellonian University Medical College, in Cracow, Poland.

Adult patients (> 18 years old) treated in the rheumatology outpatient clinic in Poland and diagnosed with axSpA based on ASAS classification criteria were included [4]. All consecutive patients treated from 1 January 2021 to 4 June 2024 who provided written consent were qualified for the study. No specific exclusion criteria were set because the data reflected real-world conditions. In total, 323 participants were involved.

Data were gathered during routine clinical visits, which were scheduled according to the treating physicians’ decisions. Data collection was performed using GoTreatIt® Rheuma software and included in a structured database as part of standard clinical care [28]. Data extraction was carried out on 4 June 2024, including information from the most recent available visit.

The extracted data included demographic variables (age, sex, body mass index [kg/m^2^], smoking status, and physical activity level), disease duration, and clinical variables (CRP [mg/dL]; number of tender and swollen joints; the presence, type, and number of comorbidities; and details of current treatment). Collected PRO measures encompassed the HAQ, Modified Health Assessment Questionnaire (MHAQ), MDHAQFn, MDHAQPs, SF-36, BASDAI, BASFI, fatigue, and pain catastrophizing.

To assess pain catastrophizing, we used two questions from the Coping Strategies Questionnaire: “When I have pain, it’s terrible and I think it’s never going to get better” and “When I have pain, I feel I can’t stand it anymore.” Both were rated on an NRS from 0 to 6, where 0 = “never,” 3 = “now and then,” and 6 = “always” and calculated as the mean of these two questions to derive the pain catastrophizing score.

Structured questionnaires and a review of the available documentation were used to ensure reliable data. Comorbidities were defined using standard definitions, and some were categorized into distinct subgroups (e.g., cardiovascular, respiratory, and gastrointestinal).

### Statistical analyses

Because this was a real-world study, no sample size calculation or power analysis was conducted. The sample size was determined by the data available from the observational sources.

First, after extraction, the database was assessed for outlier variables and overall data density. The distribution of the assessed variables was examined using histograms and the Shapiro–Wilk test. Depending on their characteristics, variables are presented as either number (percentage) or median (interquartile range). Missing data were reported to indicate data density. The nature of the missing data was evaluated using Little’s test for missing completely at random. A *p*-value of > 0.05 indicated that the missing data were missing completely at random, thus not biasing the results and allowing for further analysis.

Groups were categorized based on the number of comorbidities. The Mann–Whitney *U* test was used to identify statistically significant differences between groups. A multivariable linear regression (enter method) was conducted, with a specific PRO as the dependent variable, to assess the influence of the number of comorbidities on that variable. Models were adjusted for age, sex, disease duration, and ASDAS based on C-reactive protein (ASDAS-CRP). The variables included in the model were selected based on clinical judgment. Possible multicollinearity was tested by calculating the variance inflation factors, all of which were < 4, thus excluding multicollinearity. Adjusted *R*^2^, adjusted beta coefficients, and *p*-values were calculated and reported. A *p*-value of < 0.05 was considered statistically significant. All *p*-values were two-sided. All analyses were performed using IBM SPSS Statistics version 29.0.0.0.

## Results

There were 323 participants included in the study, 44.0% of whom were females. Their median age was 41.0 (33.0–51.0) years, and the median disease duration was 5.4 (2.3–11.7) years. The BASDAI and ASDAS-CRP scores were 2.1 (1.1–3.8) and 1.3 (0.9–2.0), respectively, indicating low disease activity. The mean body mass index was 25.4 kg/m^2^. Nearly three-quarters of the participants were HLA-B27-positive, and > 15% were current cigarette smokers. In terms of treatment, 52.3% of the patients used non-steroidal anti-inflammatory drugs, 64.7% were on biologic DMARDs, and 7.4% were on targeted synthetic DMARDs. The patients’ basic characteristics are presented in Table 1.

**Table 1:** Demographics, clinical data including pharmacotherapy, and patient-reported questionnaire results from 323 participants with axial spondyloarthritis

	Categories	Results	Missing data
Demographics	Age (years)	41.0 (33.0–51.0)	0.0%
	Women	142 (44.0%)	0.0%
	BMI (kg/m^2^)	25.4 (23.4–28.7)	4.6%
	Cigarette smoker (currently)	42 (15.8%)	17.6%
	Physical exercise > 1 h/week	143 (44.3%)	9.3%
	Years of education	15.0 (12.0–17.0)	23.8%
Clinical information	Symptom duration	11.9 (6.5–19.8)	6.2%
	Disease duration	5.4 (2.3–11.7)	9.6%
	HLA-B27–positive	236 (73.1%)	12.1%
	ESR	9.0 (5.0–16.0)	24.8%
	CRP	1.0 (1.0–4.0)	22.0%
	ASDAS-CRP	1.3 (0.9–2.0)	23.5%
Patient-reported outcomes	Pain VAS	20.0 (9.5–40.0)	9.3%
	Fatigue VAS	30.0 (15.0–53.3)	9.0%
	HAQ	0.4 (0.0–0.9)	11.8%
	MHAQ	0.1 (0.0–0.5)	9.0%
	MDHAQ Functional (0–3)	0.0 (0.0–1.1)	12.1%
	MDHAQ Psychological (0–3)	0.8 (0.3–1.0)	12.1%
	BASFI	1.4 (0.5–3.6)	11.8%
	BASDAI	2.1 (1.1–3.8)	2.8%
Current treatment	NSAIDs	169 (52.3%)	0.0%
	Glucocorticoids	7 (2.2%)	0.0%
	csDMARDs	23 (7.1%)	0.0%
	tsDMARDs	24 (7.4%)	0.0%
	bDMARDs	209 (64.7%)	0.0%

Data are presented as *n* (%) or median (interquartile range)

*ASDAS* Ankylosing Spondylitis Disease Activity Score, *bDMARDs* biological disease-modifying antirheumatic drugs, *BASDAI* Bath Ankylosing Spondylitis Disease Activity Index, *BASFI* Bath Ankylosing Spondylitis Functional Index, *BMI* body mass index, *CRP* C-reactive protein, *csDMARDs* conventional synthetic disease-modifying antirheumatic drugs, *ESR* erythrocyte sedimentation rate, *HAQ* Health Assessment Questionnaire, *MDHAQ* Multi-dimensional Health Assessment Questionnaire, *MHAQ* Modified Health Assessment Questionnaire, *NSAIDs* non-steroidal anti-inflammatory drugs, *tsDMARDs* targeted synthetic disease-modifying anti-rheumatic drugs, *VAS* visual analogue scale

The median number of comorbidities per individual was 1.0 (0.0–2.0). The most prevalent comorbidities were hypertension (22.0%), hypercholesterolemia (15.8%), obesity (15.6%), thyroid disease (11.8%), and gastrointestinal disorders (11.8%). All categories of comorbidities are presented in Table 2. Multimorbidity was reported in 206 (63.8%) of all patients included in the study. Most individuals with multimorbidity had one or two additional diseases (excluding axSpA). Extended multimorbidity, defined as the presence of two or more chronic diseases apart from axSpA in one individual, was observed in 125 (38.7%) patients. The maximum number of comorbidities in a single patient was seven.

**Table 2:** Detailed comorbidity characteristics of patients with axial spondyloarthritis

		Total	Missing data
Median number of comorbidities		1.0 (0.0–2.0)	0.0%
Multimorbidity		206 (63.8%)	0.0%
Number of comorbidities	0	117 (36.2%)	0.0%
	1	81 (25.1%)	
	2	61 (18.9%)	
	3	31 (9.6%)	
	4	21 (6.5%)	
	5	7 (2.2%)	
	6	3 (0.9%)	
	7	2 (0.6%)	
Cardiovascular disease		17 (5.3%)	0.0%
Hypertension		71 (22.0%)	0.0%
Obesity		48 (15.6%)	4.6%
Hypercholesterolemia		51 (15.8%)	0.0%
Diabetes mellitus		12 (3.7%)	0.0%
Respiratory disease		23 (7.1%)	0.0%
Thyroid disease		38 (11.8%)	0.0%
Gastrointestinal disease		38 (11.8%)	0.0%
Liver disease		6 (1.9%)	0.0%
Kidney disease		6 (1.9%)	0.0%
Urogenital disease		7 (2.2%)	0.0%
Cancer		13 (4.0%)	0.0%
Psychiatric disease		20 (6.2%)	0.0%
Neurological disease		8 (2.5%)	0.0%
Osteoporosis		30 (9.3%)	0.0%
Fibromyalgia		27 (8.4%)	0.0%
Osteoarthritis		13 (4.0%)	0.0%
Dermatological disease		8 (2.5%)	0.0%
Ear-nose-throat disease		7 (2.2%)	0.0%

Data are presented as *n* (%)

The median pain VAS score was 20.0 (9.5–40.0), while the median fatigue VAS score was 30.0 (15.0–53.3). The HAQ, MDHAQFn, MDHAQPs, and BASFI scores were 0.4 (0.0–0.9), 0.0 (0.0–1.1), 0.8 (0.3–1.0), and 1.4 (0.5–3.6), respectively; these are presented in Table 1. The results achieved in particular domains of the SF-36, reflecting health-related quality of life, are displayed in Table 3. The lowest SF-36 domain scores were observed for GH and VT, whereas the highest scores were noted for RE. The differences in the distinct categories of the SF-36 questionnaire between subgroups with and without multimorbidity are presented in Table 3. Statistically significant results were revealed for MH and VT (higher in the multimorbidity group) and for PF (higher in the no multimorbidity group than in the extended multimorbidity group). Extended multimorbidity was associated with higher HAQ scores (0.5 [0.1–1.0] vs. 0.3 [0.0–0.8], *p* < 0.01) and MDHAQ scores (0.3 [0.0–0.6] vs. 0.1 [0.0–0.5], *p* = 0.03). A statistically significant difference in the BASDAI score was observed only for patients with three or more comorbidities versus patients with no comorbidities (2.8 [1.7–4.3] vs. 1.9 [0.9–3.6], *p* < 0.01). The detailed results are displayed in Table 4.

**Table 3:** Health-related quality of life assessed using SF-36, described for participants and categorized by the presence of multimorbidity

		No multimorbidity (Group 1; *N* = 117 cases)	Multimorbidity (axSpA + ≥ 1 comorbidity) (Group 2; *N* = 206 cases)	Extended multimorbidity (axSpA + ≥ 2 comorbidities) (Group 3; *N* = 125 cases)	*p*-value (Group 1 vs. 2)	*p*-value (Group 1 vs. 3)	Missing data
SF-36	Mental health	**60.0 (48.0–76.0)**	**68.0 (56.0–76.0)**	68.0 (60.0–76.0)	**0.04**	0.07	12.3%
	Vitality	**50.0 (40.0–60.0)**	**55.0 (40.0–65.0)**	55.0 (40.0–65.0)	**0.04**	0.11	12.3%
	Bodily pain	57.5 (35.0–75.0)	57.5 (35.0–75.0)	57.5 (35.0–69.4)	0.52	0.18	12.3%
	General health	40.0 (30.0–50.0)	40.0 (30.0–50.0	40.0 (30.0–50.0)	0.67	0.35	12.3%
	Social functioning	75.0 (50.0–87.5)	75.0 (50.0–87.5)	75.0 (50.0–87.5)	0.53	0.91	12.3%
	Physical functioning	**80.0 (55.0–95.0)**	70.0 (50.0–90.0)	**70.0 (50.0–85.0)**	0.07	** < 0.01**	12.3%
	Role limitations due to physical problems	75.0 (25.0–100.0)	50.0 (0.0–100.0)	50.0 (0.0–100.0)	0.57	0.33	12.3%
	Role limitations due to emotional problems	100.0 (33.3–100.0)	100.0 (33.3–100.0)	100 (33.0–100.0)	0.89	0.39	12.3%

Data are presented as *n* (%) or median (interquartile range). The Mann–Whitney *U* test was used to identify statistically significant differences between groups. Multimorbidity is defined as living with two or more chronic illnesses, including axSpA

*axSpA* axial spondyloarthritis, *SF-36* 36-Item Short Form Survey

**Table 4:** Comparison of patient-reported outcome measures and potential differences between groups categorized by the number of comorbidities

	No multimorbidity (Group 1; *N* = 117 cases)	Extended multimorbidity (axSpA + ≥ 2 comorbidities) (Group 2; *N* = 125 cases)	Extended multimorbidity (axSpA + ≥ 3 comorbidities) (Group 3; *N* = 64 cases)	*p*-value (Group 1 vs. 2)	*p*-value (Group 1 vs. 3)
HAQ	**0.3 (0.0–0.8)**	**0.5 (0.1–1.0)**	**0.6 (0.3–1.1)**	** < 0.01**	** < 0.01**
MHAQ	**0.1 (0.0–0.5)**	**0.3 (0.0–0.6)**	0.4 (0.0–0.6)	**0.03**	0.09
MDHAQFn	0.0 (0.0–0.9)	0.0 (0.0–1.2)	0.9 (0.0–1.2)	0.17	0.05
MDHAQPs	0.8 (0.3–1.0)	0.8 (0.3–1.0)	0.8 (0.3–1.0)	0.31	0.55
Pain catastrophizing	1.00 (0.0–2.50)	1.0 (0.0–2.5)	1.5 (0.0–2.5)	0.80	0.29
Fatigue	30.0 (13.0–53.5)	30.5 (16.0–50.0)	36 (21.0–53.5)	0.96	0.22
BASDAI	**1.9 (0.9–3.6)**	2.4 (1.2–4.0)	**2.8 (1.7–4.3)**	0.10	** < 0.01**

Data are presented as median (interquartile range). The Mann–Whitney *U* test was used to identify statistically significant differences between groups. Multimorbidity is defined as living with two or more chronic illnesses, including axSpA

*axSpA* axial spondyloarthritis, *BASDAI* Bath Ankylosing Spondylitis Disease Activity Index, *HAQ* Health Assessment Questionnaire, *MDHAQ* Multi-Dimensional Health Assessment Questionnaire, *MDHAQFn* Multi-Dimensional Health Assessment Questionnaire Functional, *MDHAQPs* Multi-Dimensional Health Assessment Questionnaire Psychological, *MHAQ* Modified Health Assessment Questionnaire

Factors influencing different PROs and components of the SF-36 questionnaire are displayed in Tables 5 and 6. Women had worse outcomes in the SF-36 domains of MH, VT, PF, and SF; higher HAQ and BASDAI scores; and greater fatigue. Older age was associated with higher HAQ and MDHAQFn scores and with better results in the SF domain of SF-36. The number of comorbidities was associated only with higher scores in MH (*β* = 0.20, *p* < 0.01) and VT (*β* = 0.16, *p* = 0.02). Higher ASDAS-CRP scores were associated with worse outcomes in all PROs. Extended regression models evaluated the association of specific comorbidities with PRO measures, with results available in the supplement. Thyroid disease was associated with higher MDHAQPs scores (*β* = 0.15, *p* = 0.03) and lower VT (*β* = − 0.14, *p* = 0.04) and GH scores (*β* = − 0.15, *p* = 0.02); gastrointestinal diseases were associated with higher MDHAQPs scores (*β* = 0.24, *p* < 0.01) and higher PF scores (*β* = 0.11, *p* = 0.03); respiratory disorders were associated with a higher pain catastrophizing score (*β* = 0.16, *p* < 0.01) and lower RP (*β* = − 0.14, *p* = 0.01) and RE scores (*β* = − 0.16, *p* = 0.02); fibromyalgia was associated with a higher MDHAQFn score (*β* = 0.10, *p* = 0.04); neurological diseases were associated with higher RP scores (*β* = 0.14, *p* = 0.04); and osteoporosis was associated with higher RE scores (*β* = 0.15, *p* = 0.03).

**Table 5:** Factors influencing patient-reported outcome measures: results of adjusted regression analysis

Variables included in primary model	HAQ		MDHAQ functional		MDHAQ psychological		Pain catastrophizing		Fatigue		BASFI		BASDAI	
	*β*	*p*-value	*β*	*p*-value	*β*	*p*-value	*β*	*p*-value	*β*	*p*-value	*β*	*p*-value	*β*	*p*-value
Age	**0.27**	** < 0.01**	**0.23**	** < 0.01**	− 0.04	0.58	− 0.10	0.14	− 0.08	0.20	**0.23**	** < 0.01**	0.01	0.87
Male sex	** − 0.17**	** < 0.01**	− 0.06	0.30	− 0.17	0.09	− 0.10	0.10	** − 0.19**	** < 0.01**	− 0.10	0.06	** − 0.10**	**0.01**
Disease duration	− 0.08	0.25	** − 0.14**	**0.04**	0.06	0.49	− 0.10	0.14	− 0.06	0.33	− 0.01	0.97	− 0.06	0.15
ASDAS-CRP	**0.49**	** < 0.01**	**0.56**	** < 0.01**	**0.44**	** < 0.01**	**0.57**	** < 0.01**	**0.58**	** < 0.01**	**0.59**	** < 0.01**	**0.82**	** < 0.01**
Number of comorbidities	0.04	0.55	− 0.01	0.93	− 0.14	0.05	− 0.01	0.86	0.01	0.95	0.05	0.37	0.01	0.86
Adjusted *R*^2^	0.41		0.41		0.20		0.35		0.38		0.48		0.70	

The coefficients were derived from multivariable linear regression (entry procedure)

*ASDAS-CRP* Ankylosing Spondylitis Disease Activity Score based on C-reactive protein, *β* adjusted beta coefficient, *BASFI* Bath Ankylosing Spondylitis Functional Index, *BASDAI* Bath Ankylosing Spondylitis Disease Activity Index, *HAQ* Health Assessment Questionnaire, *MDHAQ* Multi-Dimensional Health Assessment Questionnaire

**Table 6:** Factors associated with components of the SF-36 instrument: results of adjusted regression analysis

Variables included in primary model	Mental health		Vitality		Bodily pain		General health		Social functioning		Physical functioning		Role limitations due to physical problems		Role limitations due to emotional problems	
	*β*	*p*-value	*β*	*p*-value	*β*	*p*-value	*β*	*p*-value	*β*	*p*-value	*β*	*p*-value	*β*	*p*-value	*β*	*p*-value
Age	0.04	0.60	0.09	0.22	− 0.09	0.17	0.04	0.60	**0.17**	**0.03**	− 0.30	< 0.01	− 0.08	0.28	0.03	0.75
Male sex	**0.18**	** < 0.01**	**0.15**	**0.02**	0.09	0.13	0.10	0.13	**0.21**	** < 0.01**	**0.13**	**0.01**	0.02	0.81	0.01	0.94
Disease duration	− 0.09	0.27	− 0.01	0.93	0.04	0.57	− 0.10	0.19	− 0.12	0.10	0.03	0.58	0.15	0.05	− 0.05	0.54
ASDAS-CRP	** − 0.36**	** < 0.01**	** − 0.47**	** < 0.01**	** − 0.63**	** < 0.01**	** − 0.48**	** < 0.01**	** − 0.42**	** < 0.01**	** − 0.57**	** < 0.01**	** − 0.48**	** < 0.01**	** − 0.39**	** < 0.01**
Number of comorbidities	**0.20**	** < 0.01**	**0.16**	**0.02**	0.01	0.99	0.01	0.99	0.02	0.73	− 0.07	0.19	− 0.05	0.51	0.10	0.19
Adjusted *R*^2^	**0.15**		**0.23**		**0.43**		**0.23**		**0.20**		**0.53**		**0.24**		**0.13**	

The coefficients were derived from multivariable linear regression (entry procedure)

*ASDAS-CRP* Ankylosing Spondylitis Disease Activity Score based on C-reactive protein, *β* adjusted beta coefficient, *SF-36* 36-Item Short Form Survey

## Discussion

In our study, we characterized the impact of comorbidities on PROs in patients with axSpA. The prevalence of multimorbidity in our study participants was slightly higher than in other reports [29–31] and noticeably higher than in studies concerning multimorbidity in the general population [32, 33]. We found that two or more comorbidities were associated with higher HAQ and MHAQ scores, whereas the BASDAI score was significantly higher in individuals with three or more comorbidities compared to those with no comorbidities. In many other reports, multimorbidity negatively influenced different PROs, including BASDAI and BASFI [23, 29, 30, 34, 35]. However, unlike other authors [24, 31], we did not find an association between the number of comorbidities and poorer results in PROs assessing disease activity, disability, or important symptoms such as pain or fatigue (HAQ, MDHAQFn, MDHAQPs, SF-36, BASDAI, BASFI, fatigue VAS, and pain catastrophizing) in our regression model.

With regard to quality of life, extended multimorbidity was associated with poorer scores in the PF domain (as part of the SF-36 tool) in our study. Unexpectedly, multimorbid patients had higher results in the MH and VT domains as confirmed by the regression analysis. Generally, other reports have revealed a negative impact of multimorbidity on health-related quality of life [29, 31, 34]; however, they did not analyze the particular domains of the SF-36 separately. A negative association between comorbidities and physical, but not mental, functioning has also been described in patients with rheumatoid arthritis and psoriatic arthritis [36, 37].

Our findings regarding the impact of distinct comorbidities on PROs are only partly consistent with other studies. Fibromyalgia, depression, heart failure, hypertension, chronic pulmonary disease, and peptic ulcers have been reported to be associated with higher disease activity and/or greater disability [24, 31, 38–41] as assessed by various PROs. Based on our data, thyroid disease and gastrointestinal disorders influenced psychological functioning (measured by MDHAQPs), fibromyalgia was associated with poorer functional outcomes (measured by MDHAQFn), and respiratory disorders were associated with higher pain catastrophizing scores. Quality of life, evaluated using the SF-36 tool, was worse in some domains in individuals with thyroid disease (VT, GH) and respiratory disorders (RP, RE) in our study. Some inconsistencies may result from differences in the type, number, and categorization of the analyzed comorbidities across studies. The regression model suggested that the presence of certain comorbidities may be associated with better results in specific domains of the SF-36, such as gastrointestinal disorders and PF, neurological diseases and RP, and osteoporosis and RE. These associations require confirmation in further studies; however, it is possible that patients adapt to their chronic illnesses and, by paying more attention to their health, achieve better results in certain PROs.

The sex proportion, with male predominance, and the median age of our group are generally similar to those reported in the literature [19, 24, 25, 29, 30]. The most frequent comorbidities in our population were hypertension, obesity, and hypercholesterolemia, which aligns with the data from the review and meta-analysis by Zhao et al. [23]. Many studies report comorbidities jointly for axial and peripheral SpA, with some indicating a lower frequency of comorbidities in axSpA than in peripheral SpA [25]. In our study, female sex was associated with poorer HAQ, BASDAI, fatigue scores, and SF-36 domains, including MH, VT, SF, and PF. Poorer outcomes in PROs and work productivity in women have also been observed in other reports [29, 42, 43]. The difference in the impact of comorbidities on PROs depending on sex was reported in the study by Llop et al. [44]. We also found that patients with higher ASDAS-CRP, reflecting greater disease activity, achieved inferior results across all evaluated PROs, although the cohort’s median ASDAS-CRP and BASDAI scores were within low disease activity or remission ranges.

To our knowledge, we have conducted the first study assessing the impact of comorbidities on PROs in axSpA in the Polish population. The number of included cases makes the analyses reliable. The data were collected using validated tools and during routine visits, constituting high-quality, real-world data that may contribute to an increased understanding of the association between comorbidities and PROs in axSpA. The proportions of missing data for key variables were low, reducing the risk of significant bias. A limitation of the study is the cross-sectional nature of data collection, which restricts the ability to draw conclusions about causality. Developing a prospective database for future data collection may strengthen the conclusions regarding the observed associations. Moreover, as the activity scores used in axSpA are to a large extent (ASDAS-CRP) or entirely (BASDAI), based on the patient-reported data, it is difficult to precisely assess how much they reflect the true severity of the disease and possible impact of the comorbidities, which may result in the bias in the assessment of the association between comorbidities and PROs. Additionally, reliable comparisons between different studies and registries are often challenging because of significant heterogeneity in their structure and reported PROs [45]. Despite these limitations, we consider our study a valuable contribution to understanding the association between comorbidities and PROs in axSpA.

## Conclusions

In conclusion, our data align with other reports regarding the type and prevalence of comorbidities in axSpA. Women had poorer outcomes in PROs than did men. Multimorbidity was present in nearly two-thirds of cases, with extended multimorbidity associated with poorer physical functioning, HAQ, MHAQ, and BASDAI scores, whereas multimorbidity was linked to better scores in the MH and VT domains. The impact of comorbidities on PROs in axSpA should not be overlooked; however, the detailed associations warrant further investigation.

## Supplementary Information

Below is the link to the electronic supplementary material.

Supplementary file1 (DOCX 29 KB)

## Data Availability

The data that support findings of this study are available from the corresponding author upon reasonable request.
