# Supplementary material for: Associations between comorbidities and patient-reported outcomes in axial spondyloarthritis: data from a single-center real-world cohort
Source: Clin Rheumatol. 2025 May 7;44(6):2311–20. doi: 10.1007/s10067-025-07452-6 (PMC12141145; doi:10.1007/s10067-025-07452-6)
Supplement: Supplementary file 1 — Supplementary file1 (DOCX 29 KB) [file 10067_2025_7452_MOESM1_ESM.docx]

**SUPPLEMENT**

**Table S1.** Factors influencing patient-reported outcome measures: results of adjusted analysis

| **Variables included in primary model** | **HAQ** | | **MDHAQ Functional** | | **MDHAQ Psychological** | | **Pain catastrophizing** | | **Fatigue** | |
| --- | --- | --- | --- | --- | --- | --- | --- | --- | --- | --- |
|  | β | p-value | β | p-value | β | p-value | β | p-value | β | p-value |
| **Age** | 0.25 | <0.01 | 0.23 | <0.01 |  |  | −0.09 | 0.12 | −0.11 | 0.04 |
| **Male sex** | −0.14 | <0.01 |  |  |  |  | −0.12 | 0.04 | −0.16 | <0.01 |
| **Disease duration** |  |  | −0.11 | 0.04 |  |  |  |  |  |  |
| **Global pain (VAS)** | 0.49 | <0.01 | 0.51 | <0.01 | 0.24 | <0.01 | 0.35 | <0.01 | 0.45 | <0.01 |
| **ASDAS-CRP** | 0.14 | 0.05 | 0.20 | <0.01 | 0.27 | <0.01 | 0.30 | <0.01 | 0.22 | <0.01 |
| **Number of comorbidities** |  |  |  |  | −0.28 | <0.01 | −0.17 | 0.01 |  |  |
| **Obesity** |  |  |  |  |  |  | 0.15 | 0.02 | 0.07 | 0.16 |
| **CVD** |  |  |  |  |  |  |  |  |  |  |
| **Thyroid** | 0.08 | 0.12 |  |  | 0.15 | 0.03 |  |  | 0.09 | 0.10 |
| **Diabetes** |  |  |  |  |  |  |  |  |  |  |
| **GI** |  |  |  |  | 0.24 | <0.01 |  |  |  |  |
| **Liver** |  |  |  |  |  |  |  |  |  |  |
| **Respiratory** |  |  |  |  |  |  | 0.16 | <0.01 |  |  |
| **Kidney** |  |  |  |  |  |  |  |  |  |  |
| **Urogenital** |  |  |  |  |  |  | 0.09 | 0.14 |  |  |
| **Cancer** |  |  |  |  |  |  |  |  | −0.07 | 0.16 |
| **Psychiatric** |  |  |  |  |  |  |  |  |  |  |
| **Neurologic** |  |  |  |  |  |  |  |  |  |  |
| **Osteoporosis** |  |  |  |  |  |  |  |  | −0.10 | 0.05 |
| **OA** |  |  |  |  |  |  |  |  |  |  |
| **FMS** |  |  | 0.10 | 0.04 |  |  |  |  |  |  |
| **Dermatologic** |  |  |  |  |  |  |  |  |  |  |
| **ENT** |  |  |  |  |  |  | 0.11 | 0.05 |  |  |
| **Adjusted R^2^** | 0.51 | | 0.54 | | 0.26 | | 0.41 | | 0.47 | |

**Footnote:** The coefficients were derived from multivariable linear regression with backward elimination, using an F-to-remove value of >0.20 for variable removal. The adjusted R^2^ was then calculated based on the remaining variables in the models. Gray fields indicate the variables removed from the final model due to their low significance.

**Abbreviations:** β, adjusted beta coefficient; BMI, body mass index; CVD, cardiovascular disease; ENT, ear, nose, and throat; FMS, fibromyalgia; GI, gastrointestinal disease; HAQ, Health Assessment Questionnaire; MDHAQ, Multi-Dimensional Health Assessment Questionnaire; OA, osteoarthritis.

**Table S2.** Factors associated with components of the SF-36 instrument: adjusted analysis

| **Variables included in primary model** | **Mental health** | | **Vitality** | | **Bodily pain** | | **General health** | | **Social functioning** | | **Physical functioning** | | **Role limitations due to physical problems** | | **Role limitations due to emotional problems** | |
| --- | --- | --- | --- | --- | --- | --- | --- | --- | --- | --- | --- | --- | --- | --- | --- | --- |
|  | β | p-value | β | p-value | β | p-value | β | p-value | β | p-value | β | p-value | β | p-value | β | p-value |
| **Age** | 0.14 | 0.05 | 0.12 | 0.08 | −0.09 | 0.05 |  |  | 0.18 | <0.01 | −0.28 | <0.01 |  |  |  |  |
| **Male sex** |  |  |  |  |  |  |  |  | 0.18 | <0.01 | 0.12 | <0.01 |  |  |  |  |
| **Disease duration** | −0.11 | 0.12 |  |  |  |  | −0.12 | 0.05 | −0.19 | 0.01 |  |  |  |  | −0.10 | 0.14 |
| **Global pain (VAS)** | −0.14 | 0.14 | −0.14 | 0.13 | −0.52 | <0.01 | −0.22 | 0.01 | −0.20 | 0.03 | −0.42 | <0.01 | −0.59 | <  0.01 | −0.20 | 0.04 |
| **ASDAS-CRP** | −0.26 | <0.01 | −0.36 | <0.01 | −0.25 | <0.01 | −0.33 | <0.01 | <0.30 | <0.01 | −0.25 | <0.01 |  |  | −0.23 | 0.02 |
| **Number of comorbidities** | 0.35 | <0.01 | 0.30 | <0.01 |  |  |  |  |  |  | −0.15 | <0.01 |  |  | 0.15 | 0.06 |
| **Obesity** |  |  |  |  |  |  |  |  |  |  |  |  | −0.09 | 0.10 |  |  |
| **CVD** |  |  |  |  |  |  |  |  |  |  |  |  |  |  |  |  |
| **Thyroid** | −0.11 | 0.15 | −0.14 | 0.04 |  |  | −0.15 | 0.02 |  |  |  |  |  |  |  |  |
| **Diabetes** |  |  | −0.11 | 0.14 |  |  |  |  |  |  |  |  |  |  |  |  |
| **GI** | −0.10 | 0.19 |  |  |  |  | 0.12 | 0.06 |  |  | 0.11 | 0.03 |  |  | −0.12 | 0.11 |
| **Liver** |  |  | 0.08 | 0.19 |  |  |  |  | 0.11 | 0.08 |  |  |  |  | 0.13 | 0.05 |
| **Respiratory** | −0.12 | 0.08 |  |  |  |  |  |  |  |  |  |  | −0.14 | 0.01 | −0.16 | 0.02 |
| **Kidney** |  |  |  |  |  |  |  |  |  |  |  |  |  |  |  |  |
| **Urogenital** | −0.15 | 0.09 |  |  |  |  |  |  | −0.10 | 0.11 | 0.06 | 0.19 |  |  |  |  |
| **Cancer** |  |  |  |  |  |  |  |  |  |  |  |  |  |  |  |  |
| **Psychiatric** |  |  |  |  |  |  |  |  |  |  |  |  |  |  |  |  |
| **Neurologic** |  |  |  |  |  |  |  |  | 0.10 | 0.11 | 0.08 | 0.07 | 0.14 | 0.04 |  |  |
| **Osteoporosis** |  |  |  |  |  |  |  |  |  |  |  |  | 0.11 | 0.05 | 0.15 | 0.03 |
| **OA** |  |  | −0.18 | 0.07 |  |  |  |  | 0.09 | 0.19 |  |  |  |  |  |  |
| **FMS** |  |  |  |  |  |  | 0.09 | 0.12 |  |  |  |  |  |  |  |  |
| **Dermatologic** |  |  |  |  |  |  |  |  |  |  |  |  |  |  |  |  |
| **ENT** |  |  |  |  |  |  |  |  |  |  |  |  |  |  |  |  |
| **Adjusted R^2^** | **0.17** | | **0.26** | | **0.55** | | **0.28** | | **0.23** | | **0.62** | | **0.39** | | **0.19** | |

**Footnote:** The coefficients were derived from multivariable linear regression with backward elimination, using an F-to-remove value of >0.20 for variable removal. The adjusted R^2^ was then calculated based on the remaining variables in the models. Grey fields indicate the variables removed from the final model due to their low significance.

**Abbreviations:** β, adjusted beta coefficient; BMI, body mass index; CVD, cardiovascular disease; ENT, ear, nose, and throat; FMS, fibromyalgia; GI, gastrointestinal disease; OA, osteoarthritis.
